# Anti-Inflammatory Effect of Homo- and Heterodimers of Natural Enkephalinase Inhibitors in Experimental Colitis in Mice

**DOI:** 10.3390/molecules25245820

**Published:** 2020-12-10

**Authors:** Małgorzata Sobocińska, Maciej Salaga, Jakub Fichna, Elżbieta Kamysz

**Affiliations:** 1 Laboratory of Chemistry of Biological Macromolecules, Department of Molecular Biotechnology, Faculty of Chemistry, University of Gdańsk, Wita Stwosza 63, 80-308 Gdańsk, Poland; mk.sobocinska@gmail.com; 2 Department of Biochemistry, Faculty of Medicine, Medical University of Łódź, Mazowiecka 6/8, 92-215 Łódź, Poland; salaga.maciej@gmail.com (M.S.); jakub.fichna@umed.lodz.pl (J.F.)

**Keywords:** enkephalins, sialorphin, opiorphin, spinorphin, homo-/heterodimer, inflammatory bowel diseases

## Abstract

Background: the pharmacological treatment and/or maintenance of remission in inflammatory bowel diseases (IBDs) is currently one of the biggest challenges in the field of gastroenterology. Method: our aim was the synthesis of homo- and heterodimers of natural enkephalinase inhibitors (opiorphin; sialorphin; spinorphin) and the in vitro characterization of their effect on the degradation of enkephalin by neutral endopeptidase (NEP) and stability in human plasma. We investigated the in vivo heterodimer of Cys containing analogs of sialorphin and spinorphin (peptide **X**) in a mouse model of colitis. The extent of inflammation was evaluated based on the microscopic score; macroscopic score; ulcer score, colonic wall thickness, colon length and quantification of myeloperoxidase activity. Results: we showed that the homo- and heterodimerization of analogs of sialorphin, spinorphin and opiorphin containing Cys residue at the *N*-terminal position resulted in dimeric forms which in vitro exhibited higher inhibitory activity against NEP than their parent and monomeric forms. We showed that peptide **X** was more stable in human plasma than sialorphin and spinorphin. Peptide **X** exerts potent anti-inflammatory effect in the mouse model of colitis. Conclusion: we suggest that peptide **X** has the potential to become a valuable template for anti-inflammatory therapeutics for the treatment of gastrointestinal (GI) tract inflammation.

## 1. Introduction

Inflammatory bowel diseases (IBDs), which consist primarily of Crohn’s disease (CD) and ulcerative colitis (UC), are a group of idiopathic inflammatory conditions occurring in the small intestine and the colon. The etiology of IBDs has not been revealed to date, however, it is thought to be a combination of genetic, environmental and immunological factors contributing to the pathologic reaction of the immune system in the gut [1,2]. In the course of IBDs, the excessive activation of the immune system is observed, which results in an enhanced secretion of pro-inflammatory cytokines, including tumor necrosis factor alpha (TNFα), and an imbalance in the levels of pro- and anti-inflammatory factors in the tissue [3] which is responsible for the occurrence of clinical symptoms of the disease, e.g., inflammation of the intestinal mucosa or visceral pain. The major therapeutic goals in IBD patients are the control of inflammation and the treatment of clinical symptoms such as diarrhea and rectal bleeding [4].

One of the natural sources for anti-IBD drugs is the human body, which produces endogenous opioids, enkephalins. Met-enkephalin (YGGFM) and Leu-enkephalin (YGGFL) participate in the antinociception [5], the regulation of gastrointestinal motility [5], the modulation of the immune system [6,7] and affecting anti-inflammatory, hormonal and behavioral responses [8]. Since they are natural products, they are prone to fast degradation and loss of activity. Aminopeptidase N (APN), neutral endopeptidase (NEP), dipeptidyl peptidase III (DPP III) and angiotensin converting enzyme (ACE) are the major enkephalin-degrading enzymes [9,10]. These proteases are widely distributed in the human body and are significantly involved in physiological modulation and pathophysiological processes in the gastrointestinal tract [11]. The indirect stimulation of opioid receptors by the blockade of NEP and APN is a promising pharmacological strategy for the treatment of IBDs and may become of greater importance than the use of classical opioid agonists.

Rat sialorphin (QHNPR), human opiorphin (QRFSR) and bovine spinorphin (LVVYPWT) are endogenous inhibitors of enkephalin-degrading enzymes. Studies have confirmed their efficacy in blocking the activity of enkephalinase both in vitro and in vivo. It has been demonstrated that these inhibitors possess a strong analgesic, anti-inflammatory, immunological and metabolic effect, either directly or indirectly, by affecting the level of enkephalins [12,13,14,15,16,17]. The significant limitation on the use of opiorphin, sialorphin and spinorphin in systemic therapeutic applications is the rapid degradation of their peptide bonds by circulating peptidases. In addition, in sialorphin and opiorphin, there is an issue with the stability of the *N*-terminal glutamine residue, which can be converted into a cyclic structure called pyroglutamine (Glp) in the body fluids at pH < 7 [18,19]. The metabolic half-life of native opiorphin in human plasma was evaluated at 5 min [20]. Its disappearance results in part from the cyclization of Gln^1^ to Glp (16% maximum) but mainly from the hydrolytic removal of both Gln^1^ and Glp^1^ peptides by plasma amino peptidases. The small amount of opiorphin (12%) disappears because of potential complex formation [20]. Sialorphin is also sensitive to degradation in human plasma [21].

Several modifications could help improve the metabolic stability of peptides, for instance: protecting the *N*-terminal amino group by *N*-acetylation and *C*-terminal carboxyl group by amidation, the selective substitution of amino acids with non-natural amino acids, the replacement of the *N*- and *C*-terminal amino acid residues by their respective d-enantiomer or *β*-amino acid, designing pseudo peptides or creating dimeric forms. The addition of a Cys residue at the *N*-terminal position of opiorphin led to an increase in inhibitory potency toward human APN and NEP of 10- and 5-fold, respectively, compared to the parent compound [20]. These data encourage to create dimeric forms with a disulfide bond connecting the Cys residue of each peptide chain that can be metabolically more resistant to peptidase degradation and can be more stable during storage than their monomeric forms. It is worth noting that since protecting the *N*- and *C*-terminal groups of opiorphin, sialorphin and spinorphin by *N*-acetylation and/or *C*-amidation, led to the reduction or even to the abolition of their inhibitory activity against NEP and APN [15,20], the *C*-terminal carboxyl groups of inhibitors in their dimeric forms should be left free.

In this work, we characterized in vitro the effect of homo- and heterodimers of analogs of sialorphin, spinorphin and opiorphin containing Cys residue at the *N*-terminal position on the degradation of enkephalin by NEP and stability in human plasma. We also investigated in vivo whether the most active inhibitor selected in the in vitro studies could be a potential candidate for the treatment of intestinal inflammation.

## 2. Results and Discussion

### 2.1. Peptide Synthesis and Purification

All tested peptides **I**–**XI** were synthesized using the solid-phase method [22]. The purity of the peptides after reverse-phase high performance liquid chromatography (RP HPLC) purification was higher than 98%. The identity of all peptides was confirmed by a matrix-assisted laser desorption/ionization mass spectrometry (MALDI-TOF MS) and their calculated pseudomolecular ions values were in agreement with the theoretical ones. The sequences and physicochemical characteristics of the peptides are shown in Table 1.

### 2.2. Effect of Sialorphin, Opiorphin and Homo- and Heterodimers on Degradation of Met-Enkephalin by NEP

The designed dimers (peptides **IX**–**XI**) were sparingly soluble in Tris-HCl buffer. To improve their solubility in Tris-HCl buffer, 3% DMSO was added to the samples. The addition of 3% DMSO to the Tris-HCl buffer did not help to dissolve peptide **IX**. In the time of analysis of spinorphin (**III**) and peptide **IV**, products with the same retention time as the analyzed substrate, were created, which made determining the inhibitory activity of the investigated peptides against NEP impossible.

Degradation rates (k) and half-lives (t_1/2_) of Met-enkephalin incubated with NEP alone and in the presence of peptides **I**, **II**, **V**–**VIII**, **X**, and **XI** are presented in Table 2. The elongation of opiorphin at the *N*-terminus with the Cys residue led to the production of peptide **V** which displayed a 2-fold higher inhibitory potency against NEP as compared to that of the parent compound. Our findings are compatible with previous reports showing that the elongation of opiorphin at the *N*-terminus with Cys led to an analog which shows higher inhibitory activity against APN and NEP than the parent compound [23]. The addition of the Cys residue at the *N*-terminus of spinorphin (peptide **VI**) extended the half-life of Met-enkephalin about 3.9 times in comparison to that of the sample without any inhibitor. The dimerization of sialorphin, opiorphin and spinorphin analogs containing Cys residue at *N*-terminal parts of the molecules enabled obtaining strong inhibitors (peptides **VII**, **VIII**, **X**, and **XI**). Received homo- (**VII**, **VIII**) and heterodimers (**X**, **XI**) extended the half-life of Met-enkephalin by about 5.3 (peptide **VII**), 16.7 (peptide **VIII**), 7.8 (peptide **X**) and 22.6 (peptide **XI**) times in comparison to that of the sample without inhibitor.

### 2.3. Stability in Human Plasma

**Figure 1:**
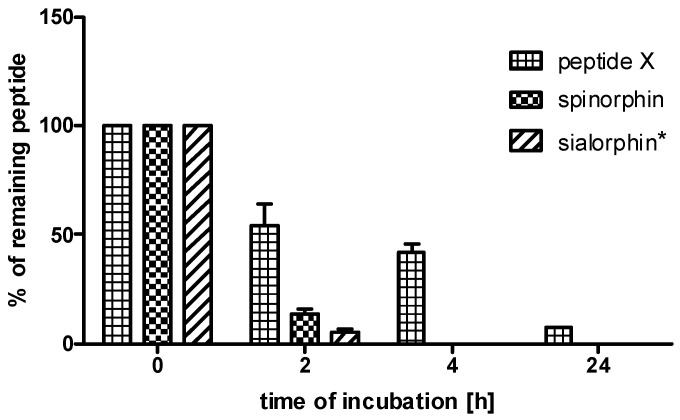
The stabilities of sialorphin, spinorphin and their analog (peptide **X**) in human plasma. The means of the % of remaining peptide with a standard deviation are presented. * data published in Sobocinska et al. [21].

Sialorphin, spinorphin and their heterodimer (peptide **X**) were incubated in the human plasma to determine their stability. In stability assays in human plasma, peptide **X** was included because this compound was selected for in vivo assays of the anti-inflammatory activity. The combination of sialorphin and spinorphin analogs in a heterodimer resulted in peptide **X**, which exhibited increased stability in human plasma. As shown in Figure 1, peptide **X** displayed greater stability in plasma than did sialorphin and spinorphin. After 2 h of incubation, the levels of peptide **X** were, respectively, 10.3 and 4 times higher in relation to those of sialorphin and spinorphin. Sialorphin and spinorphin after 4 h of incubation in human plasma were completely decomposed, while as much as 42% of peptide **X** was detected. After 24 h, the level of peptide **X** dropped to 8%. This research showed that the combination of the sialorphin analog (peptide **IV**) and the spinorphin analog (peptide **VI**) in the heterodimer (peptide **X**) would be effective in the protection of the peptide against enzymatic degradation in plasma.

### 2.4. Anti-Inflammatory Effect in TNBS-Induced Colitis

**Figure 2:**
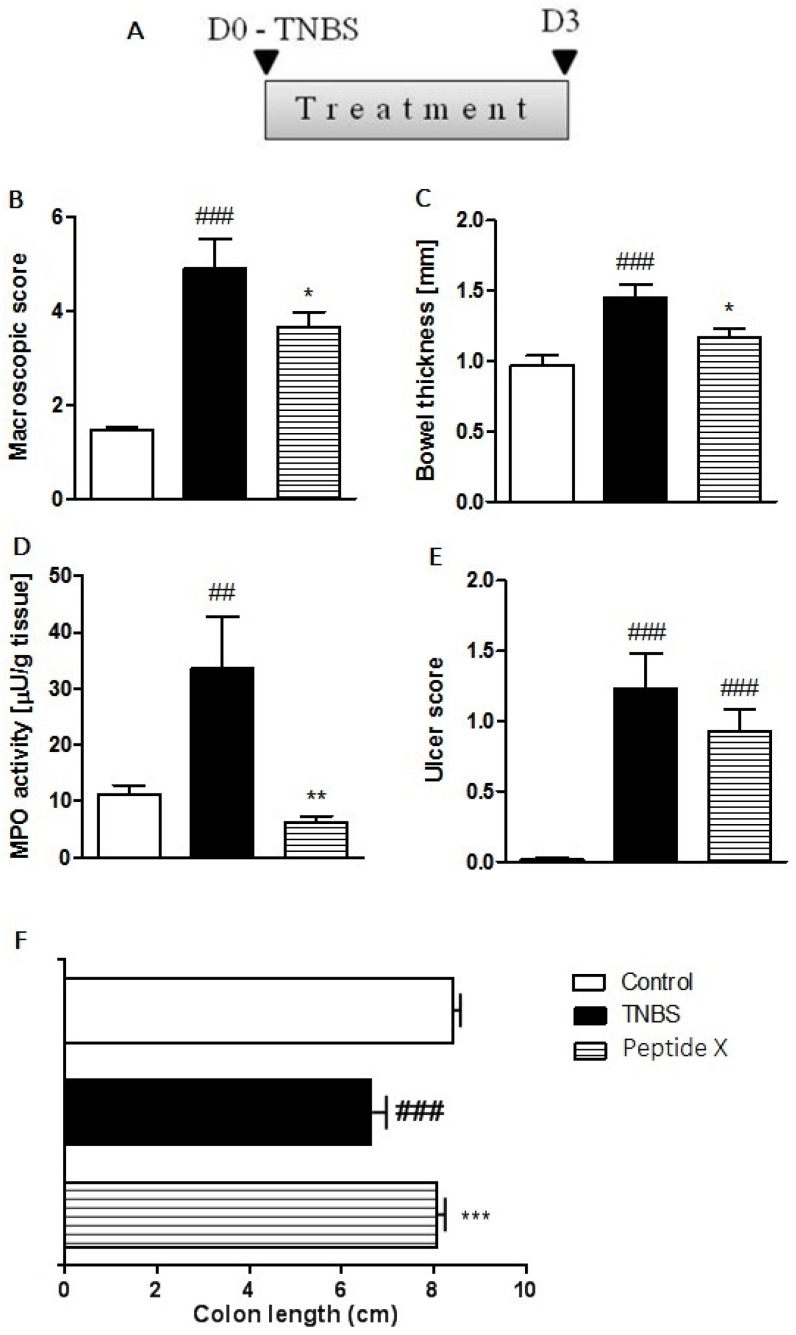
Peptide **X** injected i.p., twice daily over 3 days at the dose of 1 mg/kg attenuated TNBS-induced colitis in mice. (**A**) A scheme illustrating the protocol and treatment regimen used in this experiment. Figure shows data for the macroscopic score (**B**), bowel thickness (**C**), MPO activity (**D**), ulcer score (**E**) and colon length (**F**). * *p* < 0.05 and ** *p* < 0.01, *** *p* < 0.001 as compared with TNBS-treated animals, ^##^ *p* < 0.01, ^###^ *p* < 0.001 as compared with the control mice. Data represent the mean ± SEM of 7–9 mice per group. MPO: myeloperoxidase; TNBS: trinitrobenzenesulfonic acid.

Due to the lack of a anti-inflammatory effect in opiorphin and its analog Pal-KKQRFSR in the mouse model of trinitrobenzenesulfonic acid (TNBS)-induced colitis [15,16], only sialorphin analogs were considered for in vivo testing in the current study. To investigate the anti-inflammatory potential of the inhibitor in the GI tract, we tested the effect of the heterodimer (peptide **X**) that demonstrated favorable properties in vitro in the degradation of Met-enkephalin by NEP, in the mouse model of TNBS-induced colitis. The intracolonic (*i.c*.) instillation of TNBS caused reproducible colitis in mice, characterized by the loss of bodyweight (data not shown), increased macroscopic colon damage scores and augmented myeloperoxidase (MPO) activity. The administration of peptide **X** (1 mg/kg, i.p., B.I.D) significantly attenuated TNBS-induced colitis (Figure 2), as demonstrated by a decreased total macroscopic score (3.65 ± 0.34 vs. 4.90 ± 0.64 for TNBS-treated mice, Figure 2B), colonic wall thickness (1.16 ± 0.07 vs. 1.45 ± 0.09 for TNBS-treated mice, Figure 2C), and MPO activity (6.32 ± 1.03 vs. 33.57 ± 9.20 for TNBS-treated mice, Figure 2D). Peptide **X** did not statistically significantly reduce the degree of ulcer scores compared to the TNBS-treated mice (0.93 ± 0.15 vs. 1.23 ± 0.25 for TNBS-treated mice, Figure 2E). The shortening of the colon was more pronounced in TNBS-treated mice than in peptide **X**-treated animals (8.09 ± 0.16 vs. 6.66 ± 0.31 for TNBS-treated mice, Figure 2F). Peptide **X** showed anti-inflammatory activity comparable to sialorphin [16] and its analog Pal-KKQHNPR [15] in terms of macroscopic score. The shortening of the colon was at the same level in Pal-KKQHNPR-treated animals as in peptide **X**-treated animals. Greater reduction in the activity of MPO was observed in peptide **X**-treated animals than in sialorphin [15] or its analog Pal-KKQHNPR [16] vs. TNBS-treated mice.

### 2.5. Histological

**Figure 3:**
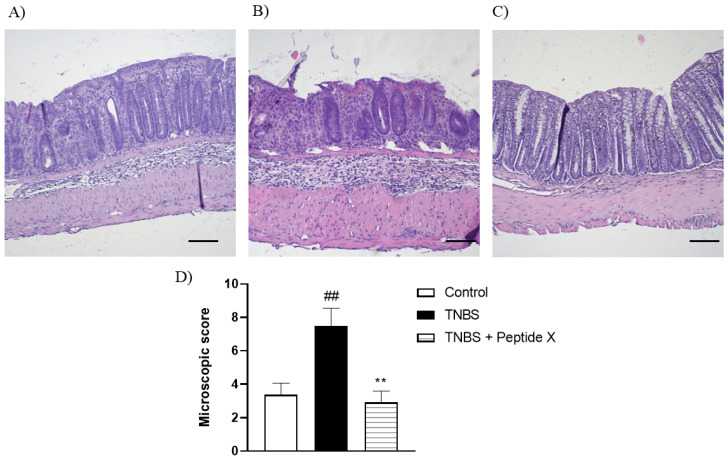
Microscopic total damage score (**D**) and representative micrographs of hematoxylin and eosin-stained sections of the distal colon from (**A**) control, (**B**) TNBS, (**C**) TNBS + peptide **X**. Scale bar = 100 μm. ^##^ *p* < 0.01, as compared to control mice, ** *p* < 0.01, vs. TNBS-treated animals. Data represent the mean ± SEM of 7–9 mice per group.

The histological evaluation of mouse colon specimens supported the macroscopic observations (Figure 3). The analysis of sections of distal colon isolated from untreated mice showed an intact epithelium, normal muscle architecture and the absence of edema (Figure 3A). Microscopic damage, characterized by a loss of mucosal architecture, the thickening of smooth muscle, the presence of crypt abscesses, as well as extensive cellular infiltration, was observed in TNBS-treated colon specimens (Figure 3B). The histological damage was significantly reduced after pretreatment with peptide **X** (1 mg/kg, Figure 3C,D).

## 3. Materials and Methods

### 3.1. Peptide Synthesis

All peptides were synthesized by the solid-phase method using Fmoc chemistry. Peptides were synthesized on 2-chlorotrityl chloride resin (loading 0.3–0.9 mmol/g, 1% DVB, 200–400 mesh, Orpegen Peptide Chemicals GmbH, Heidelberg, Germany). *N*^α^-protected amino acids and reagents used for the solid-phase synthesis were obtained from Iris Biotech GmbH (Marktredwitz, Germany). The α-amino groups of amino acids were Fmoc protected. Amino acids derivatives were as follows: Fmoc-Cys(Trt), Fmoc-Asn(Trt), Fmoc-Gln(Trt), Fmoc-His(Trt), Fmoc-Pro, Fmoc-Phe, Fmoc-Val, Fmoc-Trp, Fmoc-Arg(Pbf), Fmoc-Ser(tBu), Fmoc-Tyr(tBu), Fmoc-Thr(tBu). The first amino acid was bound to the resin according to Barlos et al. with a loading dose of 0.7 mmol/g [25]. Peptide chains were elongated in consecutive cycles of deprotection and coupling. Deprotection was performed with 20% piperidine in *N*,*N*-dimethylformamide (DMF), whereas the chain elongation was achieved with equimolar mixtures of a protected amino acid derivative, *N*,*N*′-diisopropylcarbodiimide (DIC) and 1-hydroxybenzotriazole (HOBt). Three equivalents (with respect to resin reactive groups) of these reagents were used. The completeness of each coupling reaction was monitored by the chloranil test [26]. After completing the syntheses, the peptides were cleaved from the resin simultaneously with side chain deprotection in a one-step procedure using a trifluoroacetic acid (TFA)/H_2_O/triisopropylosilane (TIS)/phenol/1,2-ethanedithiol (EDT) (93:2.5:1:1:2.5; *v*/*v*/*v*/*v*/*v*) mixture. The peptides were precipitated with ice-cold ether and lyophilized.

### 3.2. Peptide Purification and Analysis

The crude peptides were purified by HPLC on a Beckman Gold System (Beckman, Fullerton, CA, USA) controlled by the Lp-Chrome data system using an RP Kromasil-100, C8, 5 μm column (8 × 250 mm) (Knauer, Germany). The solvent system was 0.1% TFA in water (A) and 0.1% TFA in acetonitrile (B). Different linear gradients: 2–40% B over 30 min (peptides **I**, **II**, **IV** and **V**) and 10–80% B over 30 min (peptides **III** and **VI**) were applied. The mobile phase flow rate was 10 mL/min. UV detection at 214 nm was used. The purity of the synthesized peptides was checked by HPLC on a Beckman Gold System (Beckman, Irving, TX, USA) controlled by the Lp-Chrome data system, using an RP Kromasil-100, C8, 5 μm column (4.6 × 250 mm) (Knauer, Germany). The solvent system was the same as described above. A linear gradient from 2 to 40% B over 15 min (peptides **I**, **II**, **IV** and **V**) and 10 to 80% B over 15 min (peptides **III** and **VI**) with a flow rate of 1.5 mL/min and monitoring at 214 nm were applied. Fractions containing the pure peptides (>98%) were pooled and lyophilized. The mass spectrometry analysis of the synthesized compounds were carried out on MALDI-TOF mass spectrometry (a Biflex III MALDI-TOF spectrometer, Bruker Daltonics, Bremen, Germany).

### 3.3. Homo- and Heterodimers Synthesis

Homo and heterodimers were obtained as described before [27]. Briefly, peptides **IV**–**VI** (Table 1) contained cysteine residues in the sequence that allowed the formation of disulfide bridges. The Cys-linked dimers (peptides **VII**–**IX** (homodimers) and peptides **X**, **XI** (heterodimers) were prepared by dissolving the monomers in 20% (*v*/*v*) AcOH (peptide concentration: 1 mg/mL). The solution was stirred at room temperature. The free thiol groups of the peptides were oxidized by adding a dropwise 0.01 M solution of iodine in methanol. The progress of the reaction was monitored by RP-HPLC. The formation of dimers was checked by the comparison of analytical RP-HPLC profiles between monomer and dimer and confirmed by MALDI-TOF MS. The purification of dimeric forms was performed on HPLC using a linear gradient of 2–40% B over 30 min (peptides **VII** and **VIII**), 10–80% B over 30 min (peptide **IX**) and 20–70% B over 40 min (peptides **X** and **XI**). Other details of the procedure were the same as that of the monomeric forms.

### 3.4. Determination of Enkephalins Degradation Rates

Determination of Met-enkephalin degradation rates was performed, as described previously [24]. NEP was purchased from Merck (Warsaw, Poland). The solutions of Met-enkephalin, NEP and inhibitors were prepared by dissolving them in a Tris-HCl buffer (50 mM, pH 7.4). The samples were incubated over 0, 30, 60, 90 and 120 min at 37 °C and the reaction was stopped using a 1M aqueous HCl solution. In the next steps, the samples were centrifuged and the supernatants were filtered and analyzed by RP-HPLC. All measurements were performed in triplicate.

### 3.5. Stability of Sialorphin, Spinorphin and Peptide X in the Human Plasma

Plasma-stability assays were performed, as described previously [21]. The sample preparation and the analysis of peptide **I** were both performed in the same way as in our previous work [21]. Peptides **III** and **X** were analyzed by RP-HPLC using a linear gradient of 20–80% B over 20 min with the same solvent system of 0.1% TFA in water (A) and 0.1% TFA in acetonitrile (B), with a flow rate of 1.5 mL/min. All measurements were performed in triplicate.

### 3.6. Animals

Male BalbC mice (University of Łódź, Łódź, Poland) were used throughout the study. Mice (22–26 g; 6–8 weeks of age) were housed at a constant temperature (22 °C) and maintained under a 12 h light/dark cycle (lights on at 06.00) in sawdust-lined plastic cages. Chow pellets and tap water were available ad libitum. Groups of 7–9 animals were used in all in vivo experiments. All animal protocols were approved by the Medical University of Łódź Animal Care Committee (Protocol 76/ŁB640/2012) and complied with the European Communities Council Directive of 22 September 2010, the EU (2010/63/EU). All efforts were made to follow the 3R rules.

### 3.7. TNBS Model

Colitis was induced by i.c. instillation of TNBS, as described before [16]. Briefly, the mice were lightly anesthetized with 1% isoflurane (Baxter Healthcare Corp., Deerfield, IL, USA) and TNBS (in 0.1 mL of 30% ethanol in saline) was administered into the colon through a catheter. Mice were then maintained in an inclined position for 5 min and allowed to recover with food and water supplied. Control animals received vehicle alone (30% ethanol in saline). The effect of peptide **X** was evaluated as follows: colitis was induced on day 0 and peptide **X** was administered twice daily from day 0 to day 2 at the dose of 1 mg/kg intraperitoneally (i.p.) with the first treatment 30 min before the induction of colitis (Figure 2A).

### 3.8. Evaluation of Colonic Damage

Animals were sacrificed by cervical dislocation. The colon was rapidly removed, opened longitudinally, rinsed with phosphate buffered saline (PBS), and immediately examined. Macroscopic colonic damage was assessed by an established semiquantitative scoring system by adding individual scores for ulcer, colonic shortening, wall thickness, and the presence of hemorrhage, fecal blood, and diarrhea, as described before [16]. For scoring ulcer and colonic shortening, the following scale was used: ulcer, 0.5 points for each 0.5 cm; shortening of the colon, 1 point for >15%, 2 points for >25% (based on a mean length of the colon in untreated mice of 8.01 ± 0.15 cm; *n* = 8). The wall thickness was measured in millimeters, a thickness of n mm corresponding to n scoring points. The presence of hemorrhage, fecal blood, or diarrhea increased the score by 1 point for each additional feature.

### 3.9. Determination of Tissue Myeloperoxidase Activity

The method described by Sałaga et al. was used to assess the granulocyte infiltration and to quantify the myeloperoxidase (MPO) activity [16]. Briefly, 1 cm segments of colon were homogenized in hexadecyltrimethylammonium bromide (HTAB) buffer (0.5% HTAB in 50 mM potassium phosphate buffer, pH 6.0; 50 mg of tissue/mL) upon isolation and the homogenate was centrifuged for 15 min (13,200 g, 4 °C). On a 96-well plate, 200 µL of 50 mM potassium phosphate buffer (pH 6.0), containing 0.167 mg/mL of O-dianisidine hydrochloride and 0.05 µL of 1% hydrogen peroxide was added to 7 µL of the supernatant. Absorbance was measured at 450 nm (iMARK Microplate Reader, Biorad, UK). All measurements were performed in triplicate. MPO was expressed in milliunits per gram of wet tissue, 1 unit being the quantity of enzyme able to convert 1 µmol of hydrogen peroxide to water in 1 min at room temperature. Units of MPO activity per 1 min were calculated from a standard curve.

### 3.10. Histology

Segments of the distal colon were fixed in 10% neutral-buffered formalin for 24 h at 4 °C, then dehydrated in sucrose, and embedded in paraffin. Samples were sectioned at 5 μm, mounted onto slides and stained with hematoxylin and eosin. Sample examination was performed using Axio Imager A2 microscope (Carl Zeiss, Germany) and a digital imaging system consisting of a digital camera (Axiocam 506 color, Carl Zeiss, Germany) and image analysis software (Zen 2.5 blue edition, Carl Zeiss, Germany). A magnification of 100× was used to analyze the histological preparations. A microscopic total damage score was determined in a blinded fashion based on the presence (score = 1) or absence (score = 0) of goblet cell depletion, the presence (score = 1) or absence (score = 0) of crypt abscesses, the destruction of mucosal architecture (normal = 1, moderate = 2, extensive = 3), the extent of muscle thickening (normal = 1, moderate = 2, extensive = 3), and the presence and degree of cellular infiltration (normal = 1, moderate = 2, transmural = 3).

### 3.11. Statistics

Prism 5.0 (GraphPad Software Inc., La Jolla, CA, USA) was used for the statistical analysis. The data are expressed as the means ± SEM. Student t-test or one-way ANOVA followed by the Newman–Keuls post hoc test were used for the analysis, and *p*-values < 0.05 were considered statistically significant.

## 4. Conclusions

The endogenous opioid system is an attractive target in the search for new forms of IBD therapy. However, it is not an easy task to tune the opioid homeostasis through direct stimulators, like opioid agonists, due to—among others—the risk of the development of adverse side-effects upon the activation of opioid receptors in the central nervous system. Therefore, a different approach has been investigated, i.e., by tuning enzyme activity through specific blockers (opiorphin, sialorphin and spinorphin), which increase the levels of endogenous opioids, and thus stimulate opioid receptors [15,16]. We have shown in our previous work that sialorphin exerts a potent anti-inflammatory activity in the TNBS-induced acute mouse model of experimental colitis. This effect was mediated by MOR and KOR receptors, which play a major role in the regulation of the GI transit and the mucosal transport of fluids, maintaining GI homeostasis [5,16]. We also showed that sialorphin is effective in the model of chronic relapsing colitis [16]. Our observations made sialorphin a very attractive molecule for further structure-activity relationship studies in the field of IBD treatment. However, there are some limitations to its use in systemic therapeutic application, namely the proteolytic degradation of peptide bonds in this molecule and the poor stability of its *N*-terminal glutamine residue.

Dimeric forms of the peptides have the potential to be more resistant to proteases compared to monomers. In this paper, we demonstrated that the S–S bond homo- and heterodimerization of analogs of sialorphin, spinorphin and opiorphin containing Cys residue at the *N*-terminal position resulted in dimeric forms which in vitro exhibited higher inhibitory activity against NEP than their monomeric forms. The heterodimer of Cys containing analogs of sialorphin and spinorphin (peptide **X**) exerted potent anti-inflammatory effect in the mouse model of TNBS-induced colitis after systemic administration. Peptide **X** exhibited comparable anti-inflammatory activity as its endogenous parent compound (sialorphin) [16] simultaneously it was more resistant to plasma peptidase degradation.

In summary, our results suggest that the design and synthesis of novel enkephalinase inhibitors is a promising direction in the search for novel anti-IBD drugs and could become an alternative for patients for whom current treatments (NSAIDs, corticosteroids) do not provide sufficient relief.

## Figures and Tables

**Table 1:** Physicochemical properties of peptides **I**–**XI**.

Peptide	Name	Molecular Formula	HPLC t_R_(min)	[M]^+^ Calc.	[M + H]^+^ Found
**I**	Sialorphin (Gln-His-Asn-Pro-Arg)	C_26_H_42_N_12_O_8_	5.5 *	650.3	651.0
**II**	Opiorphin (Gln-Arg-Phe-Ser-Arg)	C_29_H_48_N_12_O_8_	6.7 *	692.3	693.2
**III**	Spinorphin (Leu-Val-Val-Tyr-Pro-Trp-Thr)	C_45_H_64_N_8_O_10_	8.3 **	876.5	877.4
**IV**	Cys-Gln-His-Asn-Pro-Arg	C_29_H_47_N_13_O_9_S	6.2 *	753.3	754.2
**V**	Cys-Gln-Arg-Phe-Ser-Arg	C_32_H_53_N_13_O_9_S	8.8 *	795.3	796.3
**VI**	Cys-Leu-Val-Val-Tyr-Pro-Trp-Thr	C_48_H_69_N_9_O_11_S	7.3 **	979.2	1002.4 [M + Na]^+^
**VII**	(Cys-Gln-His-Asn-Pro-Arg)_2_	C_58_H_92_N_26_O_18_S_2_	7.6 *	1504.6	1505.9
**VIII**	(Cys-Gln-Arg-Phe-Ser-Arg)_2_	C_64_H_104_N_26_O_18_S_2_	9.4 *	1588.7	1589.2
**IX**	(Cys-Leu-Val-Val-Tyr-Pro-Trp-Thr)_2_	C_96_H_136_N_18_O_22_S_2_	9.9 **	1956.9	1957.5
**X**	Cys-Gln-His-Asn-Pro-Arg │ Cys-Leu-Val-Val-Tyr-Pro-Trp-Thr	C_77_H_114_N_22_O_20_S_2_	18.6 ***	1731.0	1732.5
**XI**	Cys-Gln-Arg-Phe-Ser-Arg │ Cys-Leu-Val-Val-Tyr-Pro-Trp-Thr	C_80_H_120_N_22_O_20_S_2_	19.4 ***	1772.8	1774.2

* a linear gradient from 2 to 40% B in 15 min, ** a linear gradient from 10 to 80% B in 15 min, *** a linear gradient from 20 to 70% B in 15 min. where [A] 0.1% trifluoroacetic acid (TFA) in water, [B] 0.1% TFA in acetonitrile, column Kromasil C8 (4.6 *×* 250 mm, pore size 100 Å, particle size 5 μm), flow rate 1.5 mL/min, λ = 214 nm. The vertical lines in the sequences of peptide X and peptide XI mean the disulfide bridges between Cys residues.

**Table 2:** Degradation rates (k) and half-lives (t_1/2_) of Met-enkephalin incubated with neutral endopeptidase (NEP) alone and with peptides.

Peptide	Inhibitor	1000 × k [1/min]	t_1/2_ [min]
	Without inhibitor *	25.35 ± 1.05	27 ± 1
**I**	Sialorphin * (Gln-His-Asn-Pro-Arg)	8.84 ± 0.27 **	78 ± 2 **
**II**	Opiorphin * (Gln-Arg-Phe-Ser-Arg)	7.38 ± 0.20 **	93 ± 2 **
**V**	Cys-Gln-Arg-Phe-Ser-Arg	3.47 ± 0.12 **	199 ± 7 **
**VI**	Cys-Leu-Val-Val-Tyr-Pro-Trp-Thr	6.43 ± 0.17 **	107 ± 3 **
**VII**	(Cys-Gln-His-Asn-Pro-Arg)_2_	4.75 ± 0.08 **	145 ± 2 **
**VIII**	(Cys-Gln-Arg-Phe-Ser-Arg)_2_	1.53 ± 0.01 **	451 ± 2 **
**X**	Cys-Gln-His-Asn-Pro-Arg │ Cys-Leu-Val-Val-Tyr-Pro-Trp-Thr	3.27 ± 0.10 **	211 ± 6 **
**XI**	Cys-Gln-Arg-Phe-Ser-Arg │ Cys-Leu-Val-Val-Tyr-Pro-Trp-Thr	1.13 ± 0.04 **	612 ± 22 **

Data are the mean ± SEM. ** *p* < 0.001, compared to “without inhibitor”. * Data published in Sobocińska et al. [24]. The vertical lines in the sequences of peptide X and peptide XI mean the disulfide bridges between Cys residues.
